# Pituitary Pars Intermedia Dysfunction and Metabolic Syndrome in Donkeys

**DOI:** 10.3390/ani10122335

**Published:** 2020-12-08

**Authors:** Heidrun Gehlen, Bianca Schwarz, Claus Bartmann, Jennifer Gernhardt, Sabita D. Stöckle

**Affiliations:** 1 Equine Clinic, Veterinary Department, The Free University of Berlin, 14163 Berlin, Germany; jennifer.gernhardt@fu-berlin.de (J.G.); sabita.d.stoeckle@fu-berlin.de (S.D.S.); 2 Pferdeinternist Dr. Bianca C. Schwarz, DipECEIM, Bei der Taffingsmühle 1, 66740 Saarlouis, Germany; dr.bianca.schwarz@googlemail.com; 3 Equine Clinic, Veterinary Department, University of Giessen, 35392 Giessen, Germany; cpbartmann@gmx.de

**Keywords:** ACTH, PPID, metabolic syndrome, insulin dysregulation

## Abstract

**Simple Summary:**

Donkeys are one of the six species of the equid family. Even though they may look similar to horses, there are optical, behavioral, and physiological differences between the two species. The most important endocrine diseases in horses (equine metabolic syndrome and pituitary pars intermedia dysfunction: PPID) also exist in donkeys. The key symptoms of asinine metabolic syndrome (AMS), similar to horses, are obesity, insulin dysregulation, and laminitis. It can be diagnosed with either basal glucose and insulin concentration or dynamic tests. The intravenous glucose tolerance test and the combined glucose insulin tolerance test were evaluated for donkeys. The therapy of AMS is aimed at weight and exercise management. Donkeys suffering from PPID are often laminitic. Other authors have reported on hypertrichosis as a cardinal sign. Donkey-specific differences in shedding compared to horses have to be considered. The PPID can be diagnosed with donkey-specific reference values or dynamic testing. The dexamethasone suppression test, the thyrotropin releasing hormone (TRH) test, and the combined dexamethasone suppression/TRH test were evaluated for donkeys.

**Abstract:**

Appropriate medical care for donkeys is challenging despite being important working animals in non-industrialized countries and pets in first world countries. Although the same principles of diagnosis and therapy as in horses are commonly applied, there are differences in reference values and physiologic reaction to dynamic tests. However, donkeys seem to suffer from typical equine diseases, such as metabolic syndrome and pituitary pars intermedia dysfunction (PPID). Asinine metabolic syndrome (AMS) comprises obesity, insulin dysregulation, and laminitis. The principles of diagnosis are similar to horses. Donkey-specific reference ranges for insulin and glucose have been evaluated previously. Examinations regarding dynamic testing revealed differences in the intravenous glucose tolerance test and the combined insulin tolerance test compared to horses. The therapy of AMS is based mainly on weight loss and exercise. There are conflicting data regarding the incidence of PPID in donkeys. Laminitis and hypertrichosis were described as the main clinical signs. Species-specific and seasonal reference ranges were defined to diagnose PPID in donkeys. Furthermore, the dexamethasone suppression test, the thyrotropin releasing hormone (TRH) test and the combined dexamethasone suppression/TRH test were evaluated. Pergolide is commonly recommended for treatment.

## 1. Introduction 

The equid family comprises donkeys and zebras in addition to the horse, as well as their hybrids [1]. Donkeys are commonly used as working animals and support humans mostly in farm work and transport [2,3]. They are occasionally used for milk, leather, and meat production [3,4]. Mules and hinnies, hybrids of horses and donkeys [5,6], that are also used as working animals, are especially sure of step and, therefore, often used as pack animals in the mountains and under adverse climatic conditions [3,4,5,6,7,8]. Donkeys and their hybrids are no longer required as working animals in many European and North American countries but are kept as pets [2,9]. Consequently, the donkey population has decreased significantly [2].

Even though donkeys are important working animals in developing countries and areas that are difficult to access otherwise, and even kept as pets in first world countries [2], adequate medical care for these animals is not ensured [10,11]. Donkeys show different pharmacokinetic characteristics than the horse because of different genetic and physiological properties [11,12,13].

Endocrinologic diseases in the horse, such as pituitary pars intermedia dysfunction (PPID) and equine metabolic syndrome (EMS), are a common diagnosis. These diseases also occur in donkeys, but there are species-specific characteristics.

## 2. Asinine Metabolic Syndrome

### 2.1. Epidemiology

Donkeys are adapted to rough environmental conditions with extremes in temperature, low-quality diets, and a high workload [2]. Under these harsh conditions, the donkey developed energy-efficiency traits, with an efficiency to rapidly mobilize fat in situations of increased energy demands or when food is scarce. Due to this evolution, the donkey can be considered as a typical “easy-keeper” [14]. Therefore, obese donkeys are often encountered in developed countries if high-quality and/or calorie-rich food is provided. In the authors’ experience, owners of donkeys are often not aware that their animal is obese.

### 2.2. Clinical Signs

Donkeys have a much better feed conversion ratio than horses: a donkey requires only 57–67% of the digestible energy the pony needs compared to ponies of the same size, another point leading donkeys to be considered as typical easy keepers [15,16,17]. As fat distribution and neck morphology in donkeys differs from that in horses, a donkey-specific body condition score (BCS), and neck scoring system were developed (see Table 1 and Table 2). It is important to keep in mind that there is a possibility of calcification of fat deposits in donkeys [15]. These no longer respond to weight loss and should not be incorporated in the BCS [15]. Furthermore, donkeys may have intra-abdominal fat depots [16] that may, for example, cover the linea alba [18,19].

**Figure 1:**
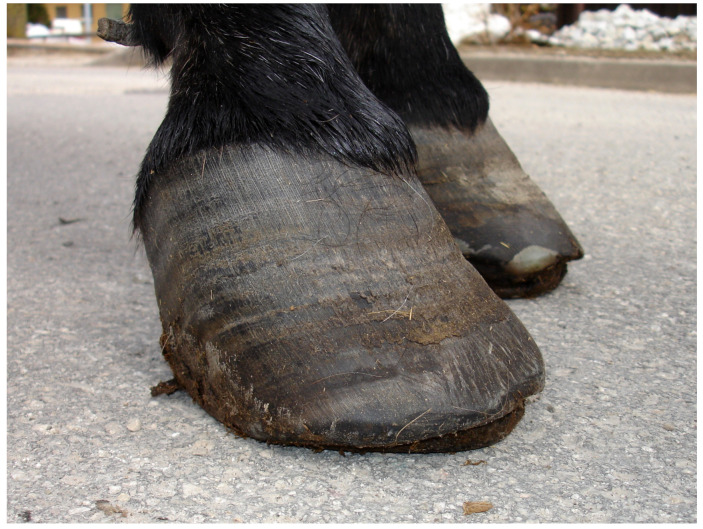
Hooves of a donkey with signs of chronic laminitis. Note the divergent growth rings.

Similar to horses, obesity and insulin resistance may contribute to laminitis [21]. Laminitis often results in irreversible changes of the anatomy of the foot, which are caused by failure of the suspensory apparatus of the distal phalanx [22,23]. These changes lead to altered biomechanics and, therefore, also to secondary pathologies and altered hoof horn production, which, in turn, leads to changes in the hoof conformation [24]. This disease causes foot pain and lameness [24]. In a study of radiological signs for laminitis in donkeys, Collins et al. [24] based the clinical diagnosis of acute laminitis on stance and gait irregularities, increased digital pulse amplitude, increased sensitivity to hoof testers in the dorsal aspect of the foot, increased hoof temperature, the presence of supracoronary depression and behavioral changes indicative of pain. Regarding the hooves themselves, the presence of divergent growth rings, widening of the white line, extensive flattening of the sole, sole hemorrhage in the region adjacent to the dorsodistal margin of the distal phalanx, distortion to the hoof capsule, dorsal concavity of the hoof wall, and/or perioplic hyperproliferation were defined as signs consistent with laminitis [24]. Radiographic signs in laminitic donkeys include a rotation, distal displacement, and/or morphometric change of the coffin bone and increases in the integument depth [24,25]. The radiological diagnosis of laminitis in donkeys, however, cannot be based on baseline data established for the horse [24]. Chronically laminitic hooves of a donkey are shown in Figure 1.

**Figure 2:**
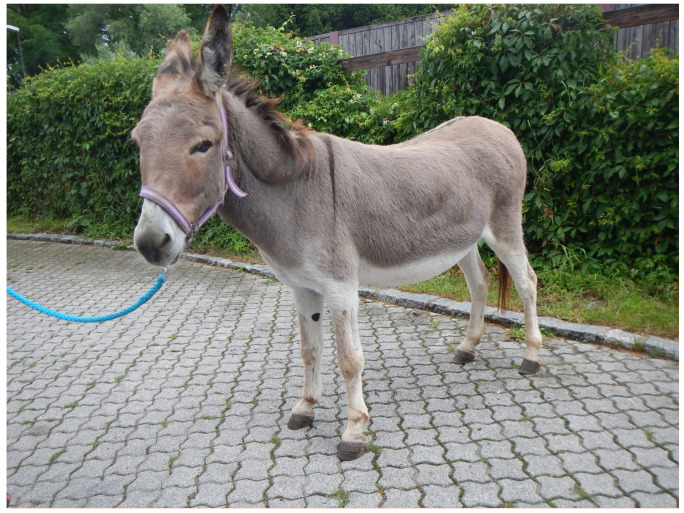
Obese donkey with a body condition score of 5.

Insulin concentrations in obese donkeys were shown to be higher when compared to moderate and thin donkeys [26]. Furthermore, an overall trend for an increasing BCS to lower insulin sensitivity has been suspected previously [26]. Additionally, there are changes in the lipid and lipoprotein metabolism in obese donkeys which may be a predisposition to hyperlipemia [27]. Compared to horses, the information available on AMS and insulin resistance in donkeys is scarce [28]. Figure 2 shows an obese donkey with a BCS of 5/5.

### 2.3. Pathogenesis

The EMS is a collection of risk factors for the development of endocrinopathic laminitis [29]. This includes obesity (regional and/or generalized) and systemic insulin dysregulation/resistance [29]. The failure of the tissues to respond to insulin adequately and/or an altered insulin clearance is defined as insulin dysregulation/resistance [29]. Genetic predisposition may be important in horses for the development of obesity and insulin dysregulation [30]. Donkeys developed energy-efficiency traits, with an efficiency to rapidly mobilize fat in situations of increased energy demands or when food is scarce as they are commonly used as working animals under harsh environmental conditions. This may predispose the animals to obesity [14].

Insulin dysregulation plays a key role in EMS and hyperinsulinemia is probably the most important pathophysiologic component of insulin dysregulation in horses [31,32]. Donkeys have a decreased insulin sensitivity [16] compared to horses and donkeys with insulin resistance often have higher insulin plasma concentrations than horses with insulin dysregulation [28]. Insulin concentrations in obese donkeys are often high, suggesting insulin dysregulation. Hyperinsulinemia in horses has previously been identified as the most probable cause of endocrinopathic laminitis [32,33,34,35], but hyperinsulinemia has not yet been identified as the direct cause of asinine endocrinopathic laminitis [17]. However, one study suggested that insulin concentrations higher than 20 µIU/mL were associated with laminitis in donkeys [36]. The clinical relevance of this or higher cutoff values to diagnose a risk of endocrinopathic laminitis remains unclear [17], especially as there are age-related variations in insulin concentration. Geriatric donkeys often show lower insulin concentrations, indicating a reduced β-cell mass or less β-cell sensitivity to glucose [20]. The gender of the animal may influence the concentration of metabolic parameters significantly [37,38]. Non-pregnant jennies have lower glucose and higher triglyceride concentrations compared to male animals [20].

### 2.4. Diagnostic Testing for Insulin Dysregulation

The diagnostic principles of insulin dysregulation in donkeys do not differ from horses. In horses, baseline insulin and glucose concentrations are recommended as a screening test for metabolic syndrome (high specificity), but the sensitivity is not adequate for ruling out EMS [39]. Newer research suggests that the cut-off values for suspected insulin dysregulation/resistance in horses might be lower than initially anticipated [40,41]. Furthermore, glucose and insulin concentrations in donkeys may be altered by previous transportation [42]. The literature suggests not providing feed containing more than 10% nonstructural carbohydrates for 6–8 h before testing a horse for insulin dysregulation/resistance [29]. For donkeys, there is no consensus on how to prepare the animals for testing, however, Mendoza et al. recommend allowing access to a flake of hay to reduce stress and avoid hyperlipemia [28]. If results of baseline testing are variable or fasting insulin is normal despite a strong clinical suspicion of EMS, dynamic testing is recommended [28]. Only a few studies have evaluated glucose-insulin dynamics in donkeys [21,37,38,43]. The combined glucose-insulin test (CGIT) and the intravenous glucose tolerance test (IVGTT) [37,43,44,45] for dynamic testing were evaluated in donkeys.

#### 2.4.1. Combined Glucose Insulin Test

**Figure 3:**
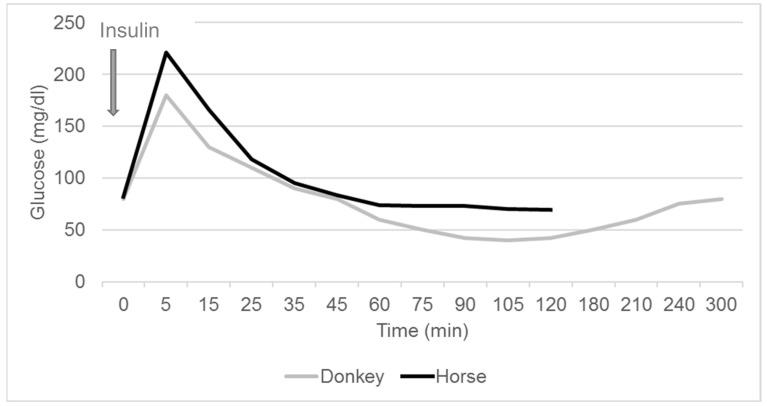
Typical combined glucose insulin test in donkeys compared to horses.

The CGIT assesses insulin sensitivity by determining the time when blood glucose concentrations return to baseline values after the simultaneous intravenous administration of dextrose/glucose (150 mg/kg, 50 % dextrose/40% glucose solution) and insulin (0.1 IU/kg) [46]. The animals need to be fasted for 6 h but are allowed free access to water. An intravenous catheter is also required. The blood glucose concentration is determined at 0, 1, 5, 15, 25, 35, 45, 60, 75, 90, 105, 120, 135, and 150 min, and insulin concentrations are measured at 0 and 45 min. The glucose curve in insulin-sensitive donkeys is shifted to the right, reaching its lowest concentration at 120 min (horse: 75 min) and it takes 240 min for glucose concentrations to return to baseline (horse: 150 min) (Figure 3) [43,47]. Insulin dysregulation in horses is suspected if the blood glucose concentration is above baseline at 45 min or the insulin concentration is greater than 100 μIU/mL 45 min after dextrose/glucose administration [43,47].

#### 2.4.2. Intravenous Glucose Tolerance Test

**Figure 4:**
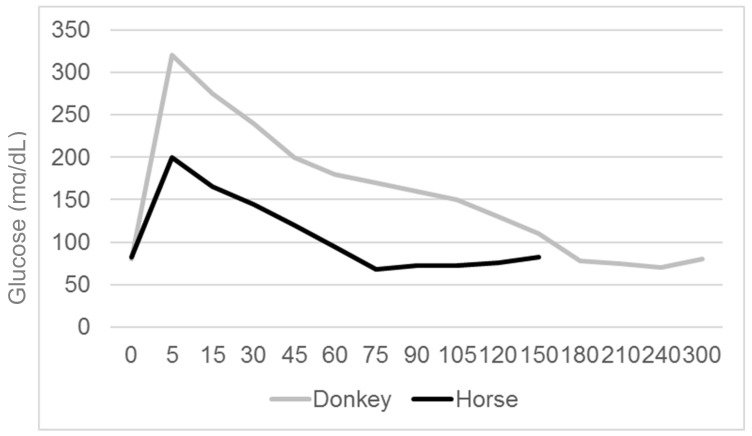
Typical intravenous glucose tolerance test in donkeys compared to horses.

The IVGTT is a method frequently used for glucose tolerance in horses [46]. The animals need to be fasted for 6–8 h for the IVGTT. Dextrose/glucose (150–300 mg/kg) is administered intravenously as a 50% or 40% solution, respectively, after collection of a baseline glucose and insulin sample. Afterwards, samples for blood glucose determination are collected every 30 min for the next 180 min. Compared to horses (150 min), donkeys also showed a right shift of the glucose curve in this test. In donkeys, the positive glucose phase lasted 160.9 ± 13.3 min (Figure 4) [43]. Furthermore, donkeys showed a negative phase in the IVGTT that is usually not present in the horse, which suggests a delayed biological efficiency of insulin [43].

Table 3 summarizes the concentrations of hormones and metabolites reported in donkeys that are typically used to evaluate metabolic disease in equids. However, it has to be mentioned that there were different methods used regarding both laboratory methods and fasting. Mendoza et al. examined 63 healthy donkeys which were mostly of Andalusian breed (9 geldings and 53 non-pregnant jennies) based on history and clinical examination as well as on hematology and blood biochemistry results [20]. In this study, the animals were starved for 12 h. Glucose and triglycerides were determined by spectrophotometry. Commercially available radioimmunoassay kits were used to determine the leptin, total adiponectin and active ghrelin concentrations [20]. These radioimmunoassay kits had been validated for horses and donkeys previously [48,49,50]. Insulin, IGF-1 and glucagon human radioimmunoassay kits were validated for donkeys by assessing the specificity, sensitivity and intra-assay precision [20], as Midgley described previously [51].

Another study evaluated the CGIT and IVGTT in ten healthy female non-pregnant Andalusian jennies. Health was based on the history, clinical examination and blood work [43]. This reference also gives two means for fasting glucose concentration. These were determined before the IVGTT and the CGIT, respectively. Glucose and triglyceride concentrations were determined by spectrophotometry. A commercial radioimmunoassay kit, that had previously been validated for donkeys [20], was used for the insulin determination. Before testing, the donkeys were housed overnight (22:00–8:00) and supplied with one flake of hay and water ad libitum.

Dugat et al. determined basal insulin and glucose concentrations in 44 mammoth donkeys and 1 miniature donkey. Animals were classified as healthy based on history and clinical examination. Of these animals, 36 were non-pregnant jennies, 5 pregnant jennies, 3 intact males, and 1 castrated male. There is neither information on feeding prior to testing nor on the laboratory methods used [52].

Gehlen et al. included data of 35–44 healthy donkeys based on history and clinical examination. The night before testing, the animals had access to grass, hay and/or straw but not to concentrates. A chemiluminescence assay was applied for insulin determination. Triglycerides and glucose were determined by photometry [53].

### 2.5. Treatment

No specific treatments have been described for donkeys suffering from ASM so far. The same principles for treating EMS generally apply [17,28]. The most important aspect of ASM therapy is clearly weight loss, which can be achieved by offering a controlled diet rich in crude fiber and low in starch [15,16]. Weight loss of 2% bodyweight per month until optimal weight and BCS is reached is targeted [28]. Despite the need for weight loss and food restriction, it has to be kept in mind that donkeys are prone to develop hyperlipemia in cases of caloric restriction/anorexia [16]. Pharmacologic treatments described for horses, for example, metformin, have not been critically evaluated in donkeys [17,28] are discussed controversially [16].

## 3. Pituitary Pars Intermedia Dysfunction

### 3.1. Epidemiology

According to McGowan, 20% of all horses, ponies, and donkeys over the age of 15 years suffer from PPID [54]. There is no gender or breed predisposition [54]. Other authors report that PPID rarely occurs in donkeys [55] and is less common than expected [56]. Cox et al. published a questionnaire study in the United Kingdom (1114 questionnaires were returned) in 2010, in which no cases of donkeys suffering from PPID were recorded [57]. Morrow et al. performed necropsies of 1444 donkeys, of which 1.9% (*n* = 27) had PPID [57]. Of these, 96.3% also had a foot disorder. This publication also reports on adrenal changes in 127/1444 donkeys (8.8%). These changes, however, are not classified further [57].

### 3.2. Clinical Signs

Signs of PPID appear to be similar in horses and donkeys. According to some authors, chronic laminitis is the cardinal symptom for PPID in donkeys [16,56], whereas others report hypertrichosis as the clinical sign observed most frequently [17,27]. However, donkeys have a physiologically longer and thicker haircoat and start shedding earlier in the autumn than horses [58]. Furthermore, shedding in donkeys takes more time in spring than it does in horses [58]. Remainders of the winter coat may still sporadically be seen in the summer [58].

Other signs, such as insulin resistance, abnormal fat distribution and muscle wasting (pot belly), that are common in horses [59] seem to also be common in donkeys suffering from PPID [17,28]. Polyuria has been reported in approximately 30% of horses suffering from PPID [60,61,62,63,64,65,66,67,68,69], but it has not yet been reported in donkeys [17]. Fertility problems, secondary infections, and lethargy have not been reported in donkeys suffering from PPID [28]. Lethargy may easily be misdiagnosed due to the donkey-specific behavior [28].

### 3.3. Pathogenesis

The pars intermedia of the pituitary gland is inhibited by dopamine provided by the hypothalamus [70]. Upon activation by dopamine, the D2 receptors mediate inhibition of proopiomelanocortin (POMC) mRNA expression and POMC-derived hormone release [71]. Degeneration of hypothalamic dopaminergic neurons in the PPID patient leads to a loss of inhibition of the melanotropes located in the pars intermedia of the pituitary gland [72]. The increased melanotrope activity results in adenoma formation and dysregulated POMC secretion [59]. The hormone precursor molecule, POMC, is synthesized in the anterior (corticotropes) and intermediate lobe (melanotropes) of the pituitary gland [73]; however, the cleavage of POMC is different between the anterior and intermediate pituitary lobes [74,75].

The POMC is split into adrenocorticotropic hormone (ACTH) and β-lipotropin in the anterior lobe. The ACTH is further cleaved in the intermediate lobe into α-melanocyte-stimulating hormone and corticotropin-like intermediate lobe peptide. Furthermore, β-lipotropin is processed to β-endorphin [74,76], of which a significantly more active form is present in the pituitary gland of PPID patients than in normal tissues [75].

The ACTH in healthy horses is only a minor (2%) cleavage product of POMC in the pars intermedia [77]. The POMC production and cleavage in the pars intermedia seems to be intact, but the post-cleavage modification of the peptides is different [73,75]. The exact consequences of these deviating concentrations remain unclear [78,79,80].

Cortisol may be responsible for some of the clinical signs associated with PPID, but many horses suffering from PPID have cortisol concentrations within or below the reference range. A recent study reported on similar total cortisol plasma concentrations in healthy and diseased horses [61,81] but found an increased free cortisol concentration (active form) in PPID patients compared to normal horses [82]. This might contribute to the clinical signs, even without an elevated total plasma cortisol concentration [82]. Similar to horses, hypercortisolemia is a rare finding in donkeys suffering from PPID [27].

### 3.4. Diagnosis

The diagnosis of PPID in donkeys relies on the same principles as in horses [17,28,59]. In horses, the TRH test is recommended for early PPID and the basal ACTH concentration for moderate to advanced PPID [83]. Plasma ACTH concentrations show seasonal changes in both horses and donkeys [53]. Furthermore, the ACTH and cortisol concentration in donkeys may be increased even after short transportation [84]. Ethylenediaminetetraacetic acid plasma is used to determine the ACTH. It is important that the sample remains cooled during overnight transportation to the laboratory [83].

The Donkey Sanctuary mentions that the same diagnostic tests can be used in the donkey for dynamic testing as those in the horse: The TRH test, the dexamethasone suppression test and the combined dexamethasone suppression/TRH test [56]. The Equine Endocrinology Group recommends firstly determining the basal ACTH concentration. If the basal ACTH concentration is within the reference range but there is a high suspicion of PPID in the patient, the TRH test is recommended for diagnostic testing [83]. At the time of writing this, performing the TRH test in autumn is not recommended because reference values for this season have not yet been established [83].

Table 4 summarizes the reference concentrations of ACTH and cortisol for donkeys provided in literature.

#### 3.4.1. TRH Test

The basal ACTH concentration is determined for the TRH test. Afterwards, the patient receives 0.5 (equids < 250 kg) or 1 mg (equids > 250 kg) TRH intravenously and the ACTH concentration is determined again 10 min after TRH administration. Transient side effects of TRH administration in horses include coughing, flehmen, and yawning [83]. A recent study evaluated the TRH test with ACTH determination in donkeys with the same criteria as in horses (positive if ACTH > 110 pg/mL 10 min post TRH administration). The test correctly identified 6/6 clinically suspect donkeys as PPID positive. The ACTH peaked at 10–20 min post TRH and returned to baseline at 30 min [85]. The TRH test can be performed after hay has been fed [86,87], but stimulation after a grain meal has not yet been examined. The Equine Endocrinology Group does not recommend testing after a grain meal [83]. The TRH test with cortisol determination is no longer recommended. Furthermore, the conduction of the TRH test in autumn is not recommended (no clinical data available) [83].

#### 3.4.2. Dexamethasone Suppression Test 

Dexamethasone administration does not suppress the cortisol release in the PPID patient as much as in the healthy equid. After determination of the basal cortisol concentration, 40 µg/kg dexamethasone is administered intramuscularly [88]. A second sample is collected 12–24 h later and the cortisol is determined. A significant decrease in cortisol concentration can be observed in the healthy patient. There is no decrease in the cortisol concentration in the PPID patient [59]. A recent study on a small number of donkeys applied the same criteria as those reported for horses in this test, i.e., cortisol concentration higher than 27.6 nmol/L at 19 h post dexamethasone administration as positive. Three out of six clinically suspect animals were identified as PPID positive in this test [85].

#### 3.4.3. Combined Dexamethasone Suppression/TRH Test

The combined dexamethasone suppression/TRH test recombines the two tests mentioned previously.

A basal sample for cortisol concentration determination is collected. Then, 40 µg/kg dexamethasone is administered intramuscularly. A second sample for cortisol determination is collected 3 h after dexamethasone administration and 1 mg TRH is administered intravenously. Several blood samples (15, 31, 45, and 60 min, 21 h) are collected afterwards [89]. A recent study evaluated the combined dexamethasone suppression/TRH test in donkeys with the same criteria as those in horses (positive if cortisol concentration >66% baseline value at 195 min post dexamethasone). This test correctly identified 67% of clinically suspect donkeys tested (4/6) as PPID-positive [85].

### 3.5. Treatment

Pergolide, a dopamine agonist derived from ergot alkaloids, is most commonly used for PPID treatment in the horse as its efficacy and safety have been demonstrated by various studies [64,90,91]. Pergolide has a high affinity to dopamine D2 receptors of the pars intermedia and, therefore, inhibits the production of POMC successfully. Based on clinical results, pergolide dosing in donkeys seems to be similar to that in horses [17,28]. A starting dose of 0.002 (0.001–0.003 mg/kg) per os once daily is recommended [16,88,92].

Pergolide is licensed for the symptomatic treatment of PPID in horses (*Prascend*®, Boehringer Ingelheim Vetmedica GmbH, Ingelheim/Germany) but not in donkeys. The side effects reported in donkeys are diarrhea, colic, depression, and anorexia [16,56,88,92].

## 4. Conclusions

The ASM and PPID in donkeys have been insufficiently studied. Diagnostic testing has recently been examined in small animal groups. Further studies are required, especially regarding the treatment of these endocrine disorders in donkeys.

## Figures and Tables

**Table 1:** Donkey-specific body condition scoring provided by the Donkey Sanctuary (https://www.thedonkeysanctuary.org.uk/sites/uk/files/2018-10/body-scoring-chart.pdf).

Condition Score	Neck and Shoulders	Withers	Ribs and Belly	Back and Loins	Hindquarters
1 Poor (Very thin) 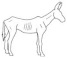 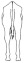	Neck thin, all bones felt easily. Neck meets shoulder abruptly, shoulder bones felt easily, angular.	Dorsal spine and withers prominent and felt easily.	Ribs can be seen from a distance and felt easily. Belly tucked up.	Backbone prominent, dorsal, and transverse processes felt easily.	Hip bones visible and felt easily (dock and pin bones). Little muscle cover. May be cavity under tail.
2 Moderate (Underweight) 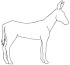 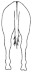	Some muscle development overlying bones. Slight step where neck meets shoulders.	Some muscle development overlying bones. Slight step where neck meets shoulders.	Ribs not visible but can be felt easily.	Dorsal and transverse processes felt with light pressure. Poor muscle development either side of midline.	Poor muscle cover on hindquarters, hip bones felt easily.
3 Ideal 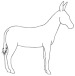 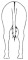	Good muscle development, bones felt under light cover of muscle/fat. Neck flows smoothly into shoulder, which is rounded.	Good cover of muscle/fat over dorsal spinous processes, withers flow smoothly into back.	Ribs just covered by light layer of fat/muscle, ribs can be felt with light pressure. Belly firm with good muscle tone and flattish outline.	Can feel individual spinous or transverse processes with pressure. Muscle development either side of midline is good.	Good muscle cover over hindquarters, hip bones rounded in appearance, can be felt with light pressure.
4 Overweight (Fat) 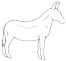 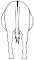	Neck thick, crest hard, shoulder covered in even fat layer.	Withers broad, bones felt with pressure.	Ribs dorsally only felt with firm pressure, ventral ribs may be felt more easily. Belly overdeveloped.	Can only feel dorsal and transverse processes with firm pressure. May have slight crease along midline.	Hindquarters rounded, bones felt only with pressure. Fat deposits evenly placed.
5 Obese (Very fat) 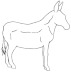 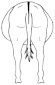	Neck thick, crest bulging with fat and may fall to one side.	Shoulder rounded and bulging with fat. Withers broad, bones felt with firm pressure.	Large, often uneven fat deposits covering dorsal and possibly ventral aspect of ribs. Ribs not palpable dorsally. Belly pendulous in depth and width.	Back broad, difficult to feel individual spinous or transverse processes. More prominent crease along midline fat pads on either side. Crease along midline, bulging fat either side.	Cannot feel hip bones, fat may overhang either side of tail head, fat often uneven and bulging.

**Table 2:** Donkey-specific neck scoring system provided by Mendoza et al. (2015) [20].

Score	Description
0 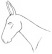	Neck thin with absence of a visible and palpable crest.
1 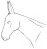	Neck still slightly thin. Crest not visible, but palpable. Normal appearance.
2 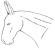	Neck moderately fatty. Noticeable crest that can be palpated from withers to poll. Patchy fat deposits can be palpated. Bony prominences cannot be felt.
3 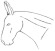	Neck thick and rounded. Crest is enlarged, thickened and hard. It is palpable from withers to poll. Crest begins to make longitudinal fat deposit to both sides of the neck. Fat deposited from the middle of the neck to withers. Crest width is increased.
4 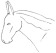	Neck thick and rounded. Crest grossly enlarged and thickened. Large fat deposits from poll to withers, forming longitudinal hard bands of fat at both neck sides that can be grasped with the hand. Crest width is grossly expanded and it cannot be grasped with one hand. Occasionally in large breeds, crest may drop to one side.

**Table 3:** Biochemical parameters and dynamic testing to evaluate metabolic disease in donkeys.

Parameter	Dynamic Testing	Mean ± SE	Mean ± SE	Mean ± SE	Mean ± SE	Mean ± SE
Glucose (mg/dL)		79.2 ± 3.5 ^a^	84.1 ± 3.7 ^a^	80.01 ± 12.25 ^d^		
Triglycerides (mg/dL)		75.3 ± 10.1^a^	66.6 ± 10.5 ^a^	58.9 ± 3.6 ^b^	66.4 ± 34.2 ^c^	75.25 ± 43.75 ^d^
Insulin (µIU/mL)		8.1 ± 0.8 ^a^	8.9 ± 0.7 ^a^	10.1 ± 0.5 ^b^	2.1 ± 2.05 ^c^	4.16 ± 3.46 ^d^
Glucagon (pg/mL)		144.2 ± 6.7 ^b^				
Leptin (ng/mL)		2.7 ± 0.3 ^b^				
Adiponectin HE (ng/mL)		458.1 ± 11.8 ^b^				
Ghrelin HE (pg/mL)		45.1 ± 1.6 ^b^				
IGF-1 (ng/mL)		234.9 ± 13.5 ^b^				
CGIT	positive phase (min)	44.1 ± 3.01 ^a^				
	negative phase (min)	255.9 ± 3.01 ^a^				
IVGTT	positive phase (min)	160.9 ± 13.3 ^a^				
	negative phase (min)	139.1 ± 13.3 ^a^				

^a^: [43], ^b^: [36], ^c^: [52], ^d^: [53], SE: Standard error of the mean; CGIT: Combined glucose–insulin test; IVGTT: Intravenous glucose tolerance test.

**Table 4:** Concentrations of ACTH and cortisol in donkeys reported in literature

Parameter	Mean ± SE	Mean ± SE	Mean ± SE
ACTH (pg/mL)	66.7 ± 20.7 ^a^ (May/June)	62.93 ± 37.4 ^b^ (Aug) 20.6 ± 14.06 ^b^ (Feb, May, Nov)	21.3–24.7 ^c^ 25.9–36.9 (Jul–Oct) ^c^ 16.5–19.5 ^c^
Cortisol (µg/dL)	4.0 ± 1.2 ^a^		

^a^: [52], no information on feeding status and laboratory method; ^b^: [53], animals had no access to concentrate feed the night before testing but unlimited access to hay, grass, and/or straw, chemiluminescence immunoassay; ^c^: [20].
